# Factors Affecting Opinion of Women Regarding the Use of Epidural Anesthesia During Labor in the Eastern Region of Saudi Arabia

**DOI:** 10.7759/cureus.32982

**Published:** 2022-12-26

**Authors:** Ilham Abdulrahman Al Mousa, Walaa Albukhaytan, Sokinah AlMusalami, Maryam Almaslami, Fatmah Alaskar, Salwa Alshaikh, Ali Aljanobe

**Affiliations:** 1 Obstetrics and Gynecology, King Faisal University, Al-Ahsa, SAU; 2 Medicine, King Faisal University, Al-Ahsa, SAU; 3 Family Medicine, Ministry of Health, Al-Ahsa Health Cluster, Al-Ahsa, SAU; 4 Medicine, Medical University of Warsaw, Warsaw, POL

**Keywords:** pregnancy, saudi arabia, labor pain, preference, awareness, epidural anesthesia

## Abstract

Objectives

Epidural anesthesia (EA) is one of the most popular and efficient techniques for labor pain relief. Women’s preferences and awareness about EA have been investigated worldwide through various studies. The level of awareness varies from region to region in Saudi Arabia. Consequently, the aim of this study is to understand the views of women regarding EA in the eastern region of Saudi Arabia and how this affects decision-making with regard to EA.

Methods

This cross-sectional study was conducted in the eastern region of Saudi Arabia from July-September, 2021, through a self-administered online questionnaire. The study included Saudi females aged 18 years and older living in the eastern region of Saudi Arabia. Data were analyzed using IBM SPSS Statistics for Windows, Version 25.0 (Released 2017; IBM Corp., Armonk, New York, United States). A p-value less than 0.05 was considered statistically significant.

Results

A total of 499 participants were included in the study. Nearly half of them were between the age of 25-35 years (46.9%, n = 234). The mean of the awareness score was 3.66 with SD = 1.491. It was observed that age was related to awareness. Women older than 45 years of age were found to be less aware. Additionally, the number of children these women had further affected their awareness; those who had four children or more tended to be less aware. The effect of regular antenatal care visits was clear, as pregnant women who maintained regular antenatal care visits showed more awareness. As for income, there was a notable increase in awareness with an increase in income. Participants who had had a previous delivery with EA were found to be more aware. Likewise, educational status also impacted their awareness. Women with a high school certificate or below were found to be less aware. Lastly, it was observed that a majority of the participants chose not to request an EA upon their next delivery (60.5%, n = 302).

Conclusion

The results demonstrated that awareness of EA in the study area is acceptable. The most important predictors for awareness about EA were age, educational status, income, number of children, regular antenatal visits, and previous delivery with EA. It was concluded that women who were aware were more likely to take EA.

## Introduction

Labor is a physiological process defined as spontaneous painful uterine contractions that are responsible for expelling the fetus from the uterus [1-2]. Labor pain is considered the worst pain for pregnant women [1]. However, labor pain is subjective and differs among women [3]. There are several methods for labor pain relief; one of them is epidural anesthesia (EA). EA is widely used and is considered to be one of the most common and effective labor pain-relieving methods [4]. EA works by numbing sensory nerves as they enter the spinal cord. The anesthetic is injected into an area of the spine known as the epidural space. Once the medication has worn off, the sensation in the affected area returns [5]. EA can effectively decrease labor pain by nearly 95% [6]. Good pain management during labor requires cooperation between obstetricians, anesthesiologists, and pregnant women.

Several studies have been conducted globally to determine the preference and awareness of women toward EA. These studies show how culture and other factors affect women’s opinions about EA. In Ethiopia, awareness about EA is low. This is because it was believed that labor pain is natural and that taking anesthesia for labor pain would be against the will of God [1]. On the other hand, EA is considered the most effective method in the United States and Canada [7]. In the Arabic region, EA is most commonly used by Palestinian [8] and Lebanese women [9].

Based on our findings, there are five studies to date regarding this topic in Saudi Arabia. In a study conducted in Khamis Mushait in 2020, results showed a low level of awareness toward EA [6]. Furthermore, in a study conducted in Najran in 2019, a lack of awareness about EA was found to exist. One of the reasons for the same was attributed to the fact that the information women had about EA stemmed from their family and friends, and not from a healthcare worker [2]. Conversely, in a study conducted in AlKhobar in 2021, with 209 participants from King Fahad University Hospital, findings revealed that most women were aware of EA for labor pain relief [10]. 

Saudi Arabia is a huge country, and each region's awareness level differs. For this reason, the primary aim of this study is to understand women’s opinions regarding EA in the eastern region of Saudi Arabia and to elucidate the factors affecting decision-making about the same

## Materials and methods

This cross-sectional study was conducted in the eastern region of Saudi Arabia from July to September, 2021, through a self-administered online questionnaire taken from Ali Alahmari [6] and Naithani [3] studies with some modifications. Approximately two minutes were needed to fulfill the questionnaire. It was translated into Arabic and divided into five sections. The first section pertained to socio-demographic data, such as age, educational status, occupation, income, marital status, pregnancy, maternity, and antenatal care. The second section focused on the labor pain experienced by women who had delivered before. The third and fourth sections explored awareness about labor pain-relieving methods and EA. The last section contained a concluding question to assess the willingness of the participants to take an EA in the next delivery. Sociodemographic data were considered predictors, while awareness level and willingness to take EA were considered as outcomes.

The sample size was determined to be 385, calculated with a margin error of 5% and a confidence level of 95%. The inclusion criteria were all Saudi females aged 18 years and above, living in the eastern region of Saudi Arabia. The exclusion criteria were females aged less than 18, or females from other areas. Additionally, those who did not complete the questionnaire were excluded from the study.

The data were analyzed using IBM SPSS Statistics for Windows, Version 25.0 (Released 2017; IBM Corp., Armonk, New York, United States). A p-value less than 0.05 was considered statistically significant. Chi-square test was used. For the purpose of knowledge interpretation, each question was assigned a score of one point. Anyone who scored less than or equal to 3 out of 6 was considered to have insufficient knowledge. Anyone who scored above 3 was considered to have enough knowledge.

Consent was obtained from all participants before they were involved in the study. All data were kept confidential, and no subject was identified by name. The study was conducted under the approval of the Institutional Review Board Committee of King Fahad Hospital, Hofuf, Saudi Arabia (approval number: 19-EP-2021).

## Results

A total of 499 participants completed the questionnaire. The study included women who were between 18 and 45 years of age. Nearly half were between 25 and 35 years of age (46.9%, n = 234). Table 1 shows the sociodemographic data of the participants.

**Table 1:** Sociodemographic data of the participants (n=499)

Sociodemographic data	Number	%
Age		
18-24 years old	124	24.8%
25-35 years old	234	46.9%
36-45 years old	96	19.2%
Older than 45 years old	45	9.0%
Educational status		
Elementary or intermediate school	19	3.8%
High school	85	17.0%
Diploma	56	11.2%
Bachelor	323	64.7%
Master or PhD	16	3.2%
Occupation		
Student	96	19.2%
Unemployed	296	59.3%
Employed	107	21.4%
Income		
Less than 5000 SR	124	24.8%
5000-10000 SR	178	35.7%
10000-15000 SR	123	24.6%
More than 15000 SR	74	14.8%
Marital status		
Married	486	97.4%
Widowed	3	0.6%
Divorced	10	2.0%
Maternity		
Yes	440	88.2%
No	59	11.8%
Number of children		
1-3 children	309	61.9%
4 children and more	124	24.8%
Pregnancy		
Yes	69	13.8%
No	430	86.2%
Antenatal care		
Regular visits	420	84.2%
Irregular visits	45	9.0%
I did not have antenatal care	34	6.8%
History of caesarean section		
Yes	137	27.5%
No	362	72.5%
Previous experience of labor pain		
Yes	394	79.0%
No (my previous delivery was a caesarean section)	38	7.6%
No (this is my first pregnancy)	67	13.4%
Previous delivery with epidural anesthesia		
Yes	113	22.6%
No	281	56.3%
Experience of epidural anesthesia complications or side effects		
Yes	40	8.0%
No	123	24.6%
In my next delivery I will request an epidural anesthesia		
Yes	197	39.5%
No	302	60.5%

**Figure 1:**
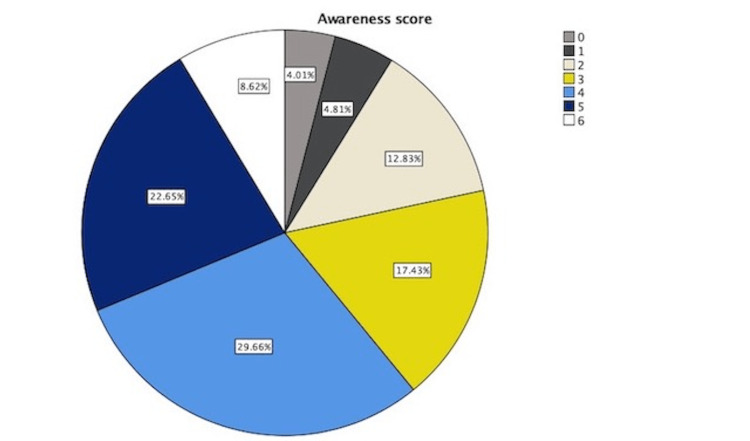
Awareness Score

Regarding the awareness score, 4.01% of participants scored 0 out of 6, only 8.62% scored 6 out of 6, about 29.66% scored 4, and 22.65% scored 5. Nearly 61% scored 4 and above, which was generally considered to be a good score. The mean of the awareness score was 3.66, with a median = 4.00 and SD = 1.491 (Figure 1).

Table 2 shows the relationships between demographic data and awareness about the EA effect. It was found that age was related to awareness (p-value of 0.008) as those older than 45 years of age were less aware. Likewise, educational status also impacted awareness (p-value of 0.002). Women with a high school certificate or below were found to be less aware. On the contrary, those with bachelor’s degrees or above were found to be more aware. According to the data, the number of children also affected awareness (p-value of 0.05) as those who had four children or more were found to be less aware, and those who had one to three children were found to be more aware. The effect of regular antenatal care visits was apparent (p-value of 0.005). Pregnant women who maintained regular antenatal care visits showed a higher level of awareness. Additionally, the study also demonstrated a significant relationship between awareness and previous delivery with EA (p-value <0.0001). It was observed that those who had tried EA before were more aware. As for income, there was a notable increase in awareness and salary. Women who had income less than 5000SR were found to be less aware. On the other hand, those who earned 10000SR or more were found to be more aware.

**Table 2:** Relationship between sociodemographic data and awareness

Sociodemographic data	Not aware	Aware	p-value
Age			
18-24 years old	43	81	0.008
25-35 years old	81	153	
36-45 years old	45	51	
Older than 45 years old	26	19	
Educational status			
Elementary or intermediate school	13	6	0.002
High school	42	43	
Diploma	22	34	
Bachelor	116	207	
Master or PhD	2	14	
Occupation			
Student	31	65	0.183
Unemployed	125	171	
Employed	39	68	
Income			
Less than 5000 SR	65	59	0.003
5000-10000 SR	67	111	
10000-15000 SR	41	82	
More than 15000 SR	22	52	
Marital status			
Married	187	299	0.131
Widowed	1	2	
Divorced	7	3	
Maternity			
Yes	172	268	1
No	23	36	
Number of children			
1-3 children	110	199	0.050
4 children and more	57	67	
Pregnancy			
Yes	18	51	0.017
No	177	253	
Antenatal care			
Regular visits	152	268	0.005
Irregular visits	27	18	
I did not have an antenatal care	16	18	
History of caesarean section			
Yes	49	88	0.357
No	146	216	
Previous delivery with epidural anesthesia			
Yes	21	92	<0.0001
No	136	145	
Experience of epidural anesthesia complications or side effects			
Yes	7	33	0.072
No	41	82	

Participants were asked whether they were willing to request EA upon their next delivery or not. It was found that age difference substantially impacted the participants’ decisions to request EA.; women between 25-35 years of age turned out to be more likely to accept EA, whereas the likelihood of refusal increased with an increase in age (p-value of 0.002). Similarly, women with four and more children tended to reject taking EA, while those with one to three children were more likely to accept EA (p-value of 0.002). Income significantly influenced the probability of taking EA. Among participants who earned less than 5000SR, about two-thirds refused to take EA, whereas over half of those who earned more than 15000SR accepted taking EA (p-value of 0.001). Women who had a previous delivery with EA had a positive attitude towards retaking it (p-value of <0.0001). Furthermore, pregnancy status failed to show any significant difference (p-value of 0.597).

Low statistical differences were spotted when evaluating educational, marital status, occupation, and maternity (p-value = 0.503, 0.573, 0.942, 0.322) respectively. For more information, refer to Table 3.

**Table 3:** Relationship between sociodemographic data and the probability of taking EA EA: epidural anesthesia

Sociodemographic data	In my next delivery I will request an EA:		p-value
	Yes	No	
Age			
18-24 years old	48	76	
25-35 years old	108	126	0.002
36-45 years old	33	63	
Older than 45 years old	8	37	
Educational status			
Elementary or intermediate school	7	12	0.503
High school	27	58	
Diploma	22	34	
Bachelor	133	190	
Master or PhD	8	8	
Occupation			
Student	39	57	0.942
Unemployed	115	181	
Employed	43	64	
Income			
Less than 5000 SR	34	90	0.001
5000-10000 SR	66	112	
10000-15000 SR	58	65	
More than 15000 SR	39	35	
Marital status			
Married	193	293	0.573
Widowed	0	3	
Divorced	4	6	
Maternity			
Yes	170	270	0.322
No	27	32	
Number of children			
1-3 children	135	174	0.002
4 children and more	34	90	
Pregnancy			
Yes	25	44	0.597
No	172	258	
Antenatal care			
Regular visits	164	256	0.391
Irregular visits	16	29	
I did not have an antenatal care	17	17	
History of caesarean section			
Yes	62	75	0.124
No	135	227	
Previous experience of labor pain			
Yes	152	242	0.547
No (my previous delivery was a caesarean section)	18	20	
Previous delivery with EA			
Yes	76	37	<0.0001
No	76	205	
Experience of EA complications or side effects			
Yes	17	23	0.094
No	69	54	

**Figure 2:**
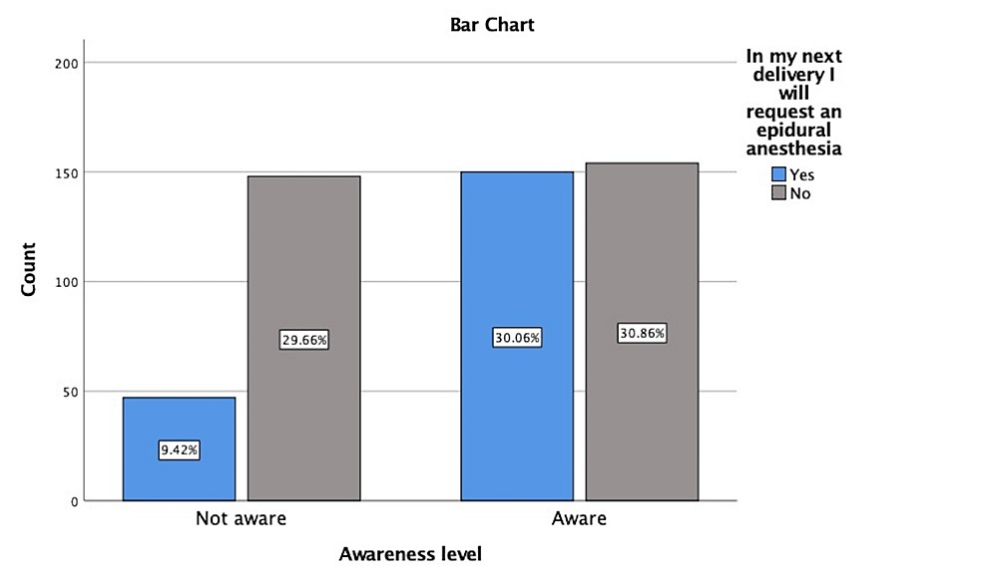
Relationship between awareness level and probability of taking EA EA: epidural anesthesia

There was a significant statistical relationship between awareness and the probability of taking EA (p-value of <0.0001) by Chi-square test (Figure 2).

## Discussion

This study aimed to assess the awareness and preference of Saudi women toward EA. The mean awareness score was 3.66, which was related to age, educational status, income, the number of children, pregnancy, antenatal care, and previous delivery with EA. Regarding preference, the data suggested a relationship between age, income, number of children, and previous delivery with EA and the probability of taking EA. 

Regarding the awareness score, more than half of the participants scored four and above (60.93%), which was considered to be a good score. This result is similar to the results of previous Saudi studies [10-11]. However, some other Saudi studies have shown a lack of awareness about EA [2,4,6]. Furthermore, in this study, it was observed that there was a significant relationship between awareness and the probability of taking EA. Those who were unaware were more likely to refuse EA in the next delivery, while those who were aware were more likely to request it. This finding is in line with the AlKhobar study [10].

An initial objective of the study was to identify the relationship between demographic data and awareness. Consequently, the study found that those older than 45 years of age were less aware. However, this result contradicts a study conducted in Nigeria, which reported that awareness was more common in older women [12]. Further, through the current study, it was found that those with a high school educational certificate or less were less aware, and those with a bachelor’s degree or higher were more aware. These results match those that have been observed in earlier studies conducted in France, Najran and Khamis Mushait in Saudi Arabia, and Nigeria [13,2,6,12]. However, they differ from some published studies, which found no relationship between education level and awareness [1,14].

Additionally, the results of this study showed that low-income women were less aware of EA. This finding is consistent with the results of Ali Alahmari, where a positive relationship between income and awareness was found to exist [6]. Regarding the relationship between awareness and the number of children, those with four or more children were found to be less aware, while those with one to three children were found to be more aware. This relation was also noticed in Nigeria [12] and can be attributed to the fact that multiparous women are less likely to take EA and, consequently, it may not be interesting for them to read about the same. Moreover, it was observed that pregnant women who maintain regular antenatal care visits showed more awareness scores. This finding is concurrent with previous studies conducted [1,6,14]. A possible explanation for this result is the education imparted by the health workers during such antenatal visits. Surprisingly, the results of this study did not show a significant difference between the group of women who had children and those who did not. However, this finding is not supported by the previous research conducted by Workie et al. in 2020, which showed a significant association between awareness of labor analgesia and parous women in comparison to nulliparous women [1].

Regarding preference, many factors affected women’s opinions toward EA. The first factor was age; women aged 25-35 were more likely to accept taking EA. This matched the results of a recent study in Alkhobar, wherein it was found that age played a role in the preference for EA; women aged 30-35 were more likely to prefer EA [10]. Furthermore, a study in Jeddah demonstrated the same result wherein women aged less than 20 or more than 35 were found less likely to prefer EA [4]. The second factor was income; those with an income of less than 5000SR were more likely to refuse to take EA, which was also significantly noticed in a previous study conducted in Lebanon [9]. Surprisingly, in the Alkhobar study, women with low income preferred EA [10]. The third factor was parity; a study in Paris reported an inverse relationship between parity and preference for EA, i.e., women with more children were less likely to choose to deliver with EA [13]. This finding was similar to the results of this study, where the number of children impacted women’s decisions to take EA, as those with four children or more were more likely to refuse to take EA. The possible explanation for this is the positive experience encountered in the previous delivery without EA and, additionally, the shorter labor time in multiparous women [13]. Moreover, in some cultures, labor pain is considered natural and medication is not prescribed to manage it [1,15], or women may want to experience a natural delivery without any analgesic methods [16]. The most significant factor examined in this study was previous experience with EA; this finding is in line with a previous study wherein it was found that women who have had a previous delivery using EA without complications had more preference toward EA [10].

Strength and limitations

The study was solely about EA. It does not apply to all types of analgesia, and it only applies to the eastern region of Saudi Arabia. Thus, it cannot be generalized to other areas. Data collection was conducted through social media. Consequently, those who do not use social media did not receive the survey.

## Conclusions

This study aimed to determine women’s awareness of EA in the eastern region of Saudi Arabia. The findings show that awareness about EA is relatively high in the study area. Age, educational status, income, pregnancy, number of children, regular antenatal visits, and previous delivery with EA were found to be the most important predictors of EA awareness. Furthermore, it was found that there was a significant relationship between awareness and the probability of taking EA i.e., women who were aware were more likely to take EA. Several factors affected their opinion and preferences to take it, such as age, income, parity, and previous delivery with EA. However, as this study was restricted to the eastern region of Saudi Arabia, it cannot be generalized. More research is needed in each region for a better understanding of attitudes toward EA, and for identifying the reasons behind the choices.
